# Dietary supplementation of *Acanthopanax senticosus* extract alleviates motor deficits in MPTP-induced Parkinson’s disease mice and its underlying mechanism

**DOI:** 10.3389/fnut.2023.1121789

**Published:** 2023-02-14

**Authors:** Jingbin Li, Yang He, Jia Fu, Yimin Wang, Xing Fan, Tian Zhong, Hui Zhou

**Affiliations:** ^1^Key Laboratory of Biotechnology and Bioresources Utilization, Ministry of Education, Institute of Plant Resources, Dalian Minzu University, Dalian, China; ^2^School of Life Sciences, Jilin University, Changchun, China; ^3^School of Health, Zhuhai College of Science and Technology, Zhuhai, China; ^4^School of Medicine, Heilongjiang University of Chinese Medicine, Harbin, China; ^5^Faculty of Medicine, Macau University of Science and Technology, Macao, Macao SAR, China

**Keywords:** *Acanthopanax senticosus* extract, MPTP, Parkinson’s disease, proteomics, signaling pathway, therapeutic mechanism

## Abstract

*Acanthopanax senticosus* extract (ASE), a dietary supplement with antifatigue, neuroprotective, and immunomodulatory properties, has been widely used due to its high polyphenol content. Our previous study showed that ASE could be used to treat Parkinson’s disease (PD) as it contains multiple monoamine oxidase B inhibitors prescribed in early PD. However, its mechanism remains ambiguous. In this study, we investigated the protective effects of ASE on MPTP-induced PD in mice and explored the underlying mechanisms of action. We found that the administration of ASE significantly improved motor coordination in mice with MPTP-induced PD. As shown by quantitative proteomic analysis, 128 proteins’ expression significantly changed in response to ASE administration, most of which were involved with Fcγ receptor-mediated phagocytosis in macrophages and monocytes signaling pathway, PI3K/AKT signaling pathway, and insulin receptor signaling pathway. Furthermore, the network analysis results showed that ASE modulates protein networks involved in regulating cellular assembly, lipid metabolism, and morphogenesis, all of which have implications for treating PD. Overall, ASE served as a potential therapeutic because it regulated multiple targets to improve motor deficits, which could lay the strong foundation for developing anti-PD dietary supplements.

## 1. Introduction

Parkinson’s disease (PD) ranks second among all neurodegenerative disorders in terms of its morbidity, and its prevalence is rising (1, 2). It has been reported that PD affects more than 6 million people worldwide, and that number may rise to 10 million by 2030 (3, 4). Its early clinical symptoms include constipation, hyposmia, cogwheel rigidity, bradykinesia, and tremors (typical triad in diagnosing PD clinically). Eventually, it leads to postural instability, freezing of gait (FOG), and ataxia during the late stages (5). Dysphonia, dysphagia, and emotionless facial expressions are also clinical features. Recent research has linked the pathogenic mechanism of PD to the degeneration of dopaminergic neurons as well as dysfunctions of astrocytes and microglia (6, 7). Nevertheless, the mechanism of PD’s pathogenesis is still only partially understood. Thus, to reduce its prevalence, new biomarkers need to be identified.

Historically used to treat a wide variety of illnesses, traditional Chinese medicine (TCM) has recently gained widespread acceptance due to its efficacy in treating a variety of conditions in clinical settings across Eastern Asian countries, especially in China (8–11). According to TCM, *Acanthopanax senticosus* extract (ASE) was used to nourish qi, fortify the spleen, tonifying the kidney, and smooth the mind (12, 13). Our previous study showed that ASE extracts could inhibit monoamine oxidase B (MAO-B) (14), making them useful for treating several disorders like Alzheimer’s disease (AD) (15), Parkinson’s disease (16), stroke (17, 18), and depression (19). Recent research indicates that ASE exerts anti-fatigue (20), neuroprotective (21), and immunomodulatory activities (22). In addition, ASE protects mice from both MPTP- and structure-mediated dysfunction as well as structural damage of mitochondria (23). However, how exactly ASE treats PD is unknown, which is why this study used proteomics technologies to try to figure it out.

Proteomics is a high-throughput and powerful approach to study the protein changes in the process of disease and it have been widely applied for the discovery of biomarkers in the pharmacology research of many major diseases, such as Human colorectal cancer (CRC) (24), hepatocellular carcinoma (HCC) (25), breast cancer (BC) (26), and Alzheimer’s disease (AD) (27). The isobaric Target for Relative and Absolute Quantification (iTRAQ)-based quantitative proteomics is the second generation of gel-free proteomic analysis that allows for the identification and quantification of proteins with a high degree of precision and consistency (28–30). It can simultaneously detect eight different samples and achieve a detailed insight into the global proteomic changes and their integrated meaning (31). This is consistent with the integrity of TCM theory and reflects the overall state of the human body under the interaction of TCM-based dietary supplements. Therefore, iTRAQ technology presents as a more appropriate research strategy to investigate the TCM’s therapeutic mechanisms.

In this study, we developed a mouse PD model by intraperitoneal injection of 1-methyl-4-phenyl-1,2,3,6-tetrahydropyridine (MPTP). We used iTRAQ-LC-MS/MS to discover biomarkers within mouse brain tissues and investigated the changes following ASE treatment. Furthermore, Ingenuity Pathway Analysis (IPA) predicted the signaling pathway and protein network regulated by ASE in treating PD, which provided novel insights into PD-related molecular alterations and assisted in identifying promising therapeutic targets for prevention, intervention, and the identification of novel potential PD biomarkers. In addition, the present work helps to understand PD pathogenesis and ASE’s therapeutic mechanism of action.

## 2. Materials and methods

### 2.1. Materials and chemicals

*Acanthopanax senticosus* root was purchased from Beijing Tongrentang Pharmacy and authenticated by Professor Zhiqiang Liu, Changchun Institute of Applied Chemistry (CIAC), Chinese Academy of Sciences, Changchun, China. High-performance liquid chromatography (HPLC)-grade methanol, acetonitrile, and formic acid were purchased from Fisher Scientific Corporation (Loughborough, UK). Other analytical-grade chemicals were supplied by Beijing Chemical Works (Beijing, China). Ultrapure water was made using the Milli-Q purification system (Billerica, MA, USA). Dimethyl sulfoxide (DMSO), Fetal bovine serum (FBS), penicillin, streptomycin, Dulbecco’s modified Eagle’s medium (DMEM), [3-(4,5-dimethylthiazol-2-yl)-2,5-diphenyl-2 Htetrazolium bromide (MTT), and Trypsin were supplied by Beijing Dingguo Changsheng Biotechnology Co., Ltd. (Beijing, China). MPTP was obtained from Shanghai Demo Pharmaceutical Technology Co., LTD. (Shanghai, China). AKT and JAK1 antibodies were purchased from Abcam (Cambridge, MA).

### 2.2. Preparation of ASE

The ASE solution was prepared by first soaking 5 kg of dried *Acanthopanax senticosus* roots in distilled water (10-fold volumes) for 0.5 hours, then heating the resultant mixture for one hour, and finally filtering the supernatants. The roots were extracted several times with water (10-fold volume). The combined extracts were then concentrated into an aqueous ASE. Following overnight precipitation with 70% ethanol, the extract was passed through the AB-8 macroporous resin column to separate the supernatant. Elution was later carried out with 30% ethanol at a flow rate of 4 BV/h and 5-fold volume. The ASE extract powder was obtained after the rotary evaporation and lyophilization of 30% ethanol eluate.

### 2.3. UPLC-MS analysis of ASE

The liquid chromatographic separation was performed with a Waters Acquity UPLC system equipped with a Waters Acquity BEH C18 1.7 μm, 2.1 (i.d.) × 50 mm column (Waters Corp., MA, USA). The sample injection volume was 5 μL and the column temperature was kept at 35°C. The gradient elution mobile phases contained 0.1% formic acid in water (phase A) and acetonitrile (phase B). The gradient elution was performed with 0.3 mL/min of flow rate as follows: 5-25% B, 0-8 min; 25-55% B, 8-14 min; 55-80% B, 14-17 min; 80-95% B, 17-18 min; 95-100% B. Tandem mass spectra were measured on a SYNAPT G2 Q-TOF HDMS (Waters MS Technologies, Manchester, UK). The mass spectrometer parameters were set as follows: ion source temperature: 100°C; m/z scan range: 100 to 1,500 Da; desolvation temperature, 250°C; cone gas (N2): 50L/h; desolvation gas (N2): 600L/h; capillary voltage: 2.5 kV; Leucine encephalin reference ions with m/z of 556.2771 (for ESI +) or 554.2615 (for ESI-) were infused during data acquisition for online calibration. MS*^E^* was applied for MS/MS analysis with a low collision energy of 6 eV and a high collision energy of 20-45 eV. LC-MS/MS data were acquired and analyzed using MassLynx 4.1 software (Waters). The compound structural elucidation was performed in reference to MassBank and ChemSpider.

### 2.4. Animal grouping and model preparation

A total of 60 C57/BL6 mice (male, 8 weeks old) weighting 200-220g were purchased from Liaoning Changsheng Biotechnology (Liaoning, China). The mice were housed in a temperature-controlled (22 ± 2°C) room with a 12 h light/dark cycle and free access to food and water. After 1 week of adaptive feeding, The mice were randomly split into 4 groups (*n* = 15) for this study: controls, models, Madopar-treated, and ASE-treated. Madopar-treated and ASE-treated mice were administrated with Madopar (100 mg kg^–1^d^–1^) and ASE (100 mL kg^–1^d^–1^, which is equivalent to 4.5 g crude drug kg^–1^d^–1^) once a day for 15 days, respectively. Mice in model and control groups were administrated with an equal amount of distilled water. The mice in all groups were administered daily for 15 days via oral gavage. The mice in the model, Madopar-treated, and ASE-treated groups received 1-methyl-4-phenyl-1,2,3,6-tetrahydropyridine (MPTP) (30 mg/kg/d) via intraperitoneal injection for 7 days starting on day 8, while the mice in the control group received saline via intraperitoneal injection. MPTP is a well-known dopaminergic neuron toxin, which causes dopaminergic neuronal loss and degeneration through enhancing oxidative stress (OS) while suppressing mitochondrial respiratory-chain complex I (32, 33). At the end of the experiment, mice were subjected to behavioral tests. Every experiment was carried out following the Guide for Care and Use of Laboratory Animals.

### 2.5. Locomotor function assessment

Four motor behavior tests were used to evaluate the locomotor function after ASE administration: the spontaneous activity test, the rotarod test, the pole-climbing test, and the hanging test.

#### 2.5.1. Spontaneous activity test

Using an open field test (OFT), the amount of spontaneous locomotory movement in mice was measured. In brief, each mouse was adapted to the environment for a 2-h period before OFT and put separately to face an identical wall of a white square box (dimension, 50 × 50 × 25 cm) in the dark for a 5-min period in a quiet environment. The number of spontaneous activities of mice in each group was recorded.

#### 2.5.2. Rotarod test

On the seventh day after the last MPTP injection, the accelerating rotarod apparatus was utilized to assess rotarod performance using a suspended rod (3 cm in diameter) at the constant accelerating rate of 20 rpm/s. After 300 s or after the mice fell off the rotarod, the test was ended.

#### 2.5.3. Pole-climbing test

Each mouse was put on top of the wooden pole (height, 50 cm; diameter, 8 mm) with a rough surface. This work later recorded the overall time needed for each animal to descend the pole (until the mouse reached the floor) and turn. Bradykinesia was reflected by the delayed or extended time necessary for completing the test.

#### 2.5.4. Hanging test

Every mouse was put onto the horizontal wire (diameter, 1.5 mm), followed by suspension 30 cm away from the ground. After that, the limb coordination of mice was detected by recording the hanging time.

### 2.6. Proteomic analysis

#### 2.6.1. Protein sampling

After the motor behavior tests, all the mice were sacrificed by cervical dislocation. The total protein from each cerebral tissue sample was homogenized on ice. After centrifugation of cell lysate for 30 min at 14,000 rpm, the supernatant was collected, and protein concentration was determined using the BCA assay.

#### 2.6.2. iTRAQ labeling

In order to reduce variation between biological samples, equal amounts of protein from each of the 7 brain tissues were mixed to generate one normalization pool. In total, 8 mixing sample pools were obtained: control group 1, control group 2, model group 1, model group 2, Madopar-treated group 1, Madopar-treated group 2, ASE-treated group 1 and ASE-treated group 2. Proteins (100 μg) from each pool were denatured, alkylated, and subjected to overnight trypsin digestion at a 1:20 enzyme/protein ratio at 37°C, followed by iTRAQ reagent tag labeling with the 8-plex iTRAQ kit according to manufacturer’s protocol. Tryptic peptides derived from the control group 1, control group 2, model group 1, model group 2, Madopar-treated group 1, Madopar-treated group 2, ASE-treated group 1 and ASE-treated group 2 were labeled as 114, 115, 116, 117, 118, 119, and 121 respectively. This labeling reaction was kept at room temperature for 1 h before mixing the labeled samples in the same ratio. The technical repeats were used to adjust experimental randomness, and proteins were screened twice (biological repeats) using the iTRAQ procedure.

#### 2.6.3. Ultra-performance liquid chromatography (UPLC) fractionation as well as LC–MS/MS analysis

The Sep-Pak Vac C18 (Waters, MA, USA) was used to process the labeled samples to remove salts. Meanwhile, peptides were fractionated using UPLC (Waters, Milford, MA, USA) (34–36). Following that, the bridged ethylene hybrid C18 column (2.1 × 50 mm, 1.7 μm) was used for analysis. Later, elution was done with a linear gradient of 2 mobile phases (solvent A consisted of ammonium formate (20 mM), pH 10, and solvent B consisted of acetonitrile, which began with 5% solvent B then increased to 35% within 16 min) at a flow rate of 600 μL/min. Absorbance (A) was measured at 214 nm, and 10 fractions were harvested.

The nano-HPLC (Eksigent Technologies) was used to separate fractions using a secondary reversed-phase analytical column (Eksigent, C18, 3 μm, 150 mm × 75 μm). After peptide separation, they were eluted from the analytical column at a flow rate of 300 nL/min using two mobile phases (solvent A: 2% acetonitrile with 0.1% FA; solvent B: 98% acetonitrile with 0.1% FA), beginning with 5% solvent B and holding for 5-min, then 5–40% B gradient within 65 min, ramping to 80% B within 1 min, 5-min holding with 90% B, ramping to 5% B within 1 min, 18-min holding with 5% B before subsequent sample loading. The eluted sample was simultaneously passed through a mass spectrometer at the 2.5 kV electrospray voltage.

For the automatic shift between MS/MS and MS acquisition, this work used the information-dependent data acquisition mode of the Q-Exactive mass spectrometer. We acquired the full-scan MS spectra at 350–1250 m/z. Altogether 25 of the high-intensity precursors were chosen in each cycle’s fragmentation, and the dynamic exclusion time was 25 s.

### 2.7. Identification, quantitation, and bioinformatics analyses

Each MS/MS sample was searched against the UniProt-mouse database with the MIS search type using the Mascot search engine (Matrix Science, London, UK; version 2.3.02). Carbamidomethyl (C) and iTRAQ 8 plex (K) were selected as fixed modifications. Later, resultant peptide hits with the maximal 5% false discovery rate (FDR) were selected. iTRAQ8plex quantification analysis was used to determine reporter ratios, with fragment and peptide mass tolerances of ± 0.1 Da and 20.0 PPM, respectively.

Biological functions together with protein interaction pathways with obvious changes were identified using IPA software (IPA software v7.1, Ingenuity System Inc., Redwood City, CA, USA; www.ingenuity.com). We used cutoffs of a fold change (FC) of 1.2 and a t-test significance level of p 0.05 from two independent replicates to classify differentially expressed proteins as either up- or down-regulated. Simultaneously, the right-tailed Fisher’s exact test was used to calculate p-values. Later, protein scores based on p-values were calculated, representing the clustering possibility of proteins identified in the network. Typically, networks with scores greater than 2 were clustered.

Statistical analysis: All data were expressed as mean ± SD and analyzed using GraphPad Prism software. The Student’s t-test was used to analyze the statistical significance of the data between two groups. A probability of p < 0.05 was considered to reflect statistical significance.

## 3. Results

### 3.1. Composition analysis of ASE

**FIGURE 1:**
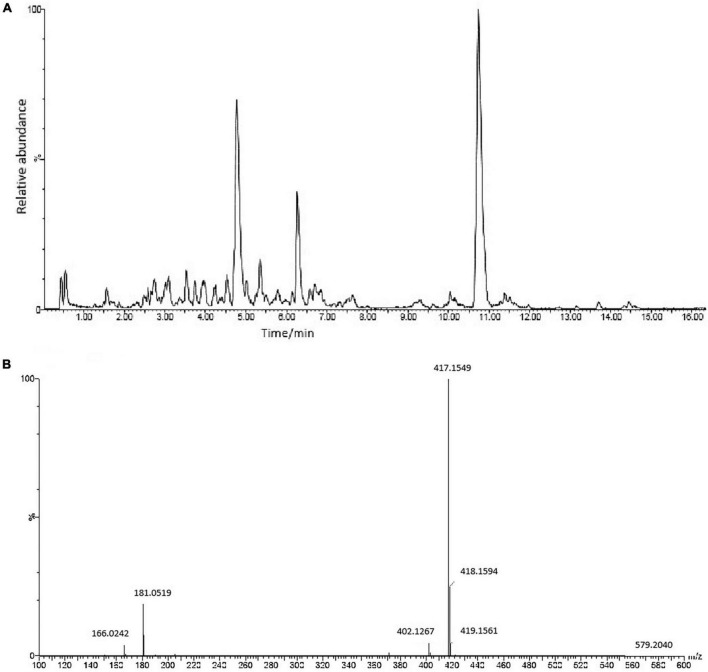
**(A)** The chemical composition of ASE identified by UPLC-ESI-Q-TOF-MS/MS analysis in negative ion mode. **(B)** MS/MS spectrum of Syringaresinol-4-O-β-D-glucoside.

UPLC-Q-TOF ESI MS/MS was used to interpret a mass spectral fragmentation pattern to determine ASE’s chemical makeup. Before analysis, we optimized ESI-MS parameters, like electrospray voltage, capillary temperature, and capillary voltage. Negative ion mass spectrometry provided more structural information for ASE than positive ion mass spectrometry. The ASE was well separated and detected within 16 min (Figure 1). During this study, molecular weight and retention time obtained by Q-TOF-MS, along with fragment ion data obtained by MS/MS, were used primarily in the identification process. Eighteen chemical components were identified including 5 organic acid compounds (vanillic acid, chlorogenic acid, p-coumaroyl quinic acid, dicaffeoylquinic acid, 3-O-caffeoyl feruloylquinic acid) and 13 phenolic glycosides (glucosyringic acid, ferulyl quinic acid glucoside, isofraxidin, dimethoxyl lariciresinol glucoside, pinoresinol diglucoside, pinoresinol, guaicylglycerol hydroconiferol glucoside, medioresinol glucoside, syringaresinol-4-O-β-D-glucoside, eletutheroside E, ciwujiatong glucoside, syringaresinol, trihydroxy-octadecenoic acid). According to relevant literature reports, triterpenoid saponins, lignans, coumarins and flavonoids constitute the majority of compounds found in ASE. Lignans and coumarins are also considered phenolic compounds by some researchers. Thus, phenolic compounds are the main active constituents of ASE.

### 3.2. ASE alleviates motor deficits in mice with MPTP-induced Parkinson’s disease

**FIGURE 2:**
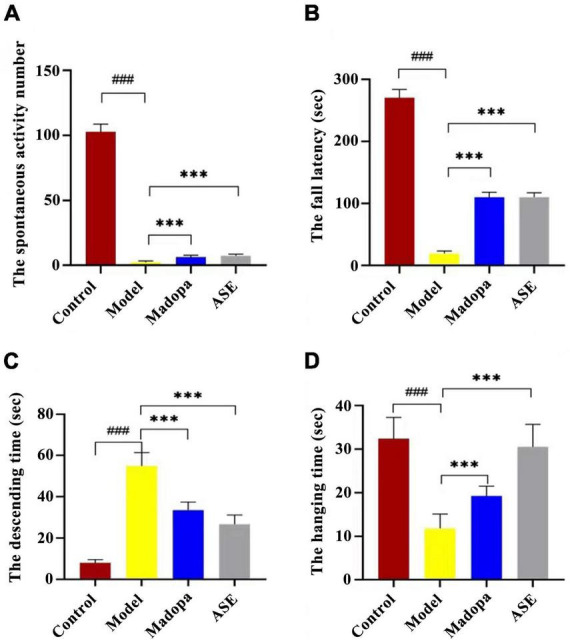
ASE treatment alleviates motor deficits in MPTP-induced Parkinson’s disease mice. **(A)** the spontaneous activity test; **(B)** the rotarod test; **(C)** the pole-climbing test; **(D)** the hanging test. The data are presented as the means ± SDs of 15 biological replicates (###*p* < 0.01, compared with the control group; ^***^p < 0.05, compared with the model group).

Behavioral tests, such as spontaneous activity, rotarod, and suspension pole-climbing tests, were used to determine how ASE suppression affected MPTP-mediated behavioral deficits (Figure 2). In the spontaneous activity test, the spontaneous activity score of the model group was significantly lower than the control group (*p* < 0.01). compared with the model group, madopa-treated and ASE-treated mice showed a significant increase in spontaneous activity. The latency to fall in the rotarod test and the hang time in the suspension test decreased in the model group compared with those in the control group. In contrast, Madopa-treated and ASE-treated mice showed a significant increase in the rotarod test and the suspension test. Similarly, the Madopa-treated and ASE-treated groups showed a significantly shorter time to descend on the pole-climbing test compared to MPTP-treated mice. These results suggest that ASE may play an anti-PD role by protecting the neurocytotoxicity of MPTP in mice, relieving some external behavioral ability, and restoring the dysregulated motor coordination seen in mice with Parkinson-like symptoms. It was further confirmed that ASE helps in both the prevention and treatment of PD.

### 3.3. ASE modulates MPTP-induced protein expression

**FIGURE 3:**
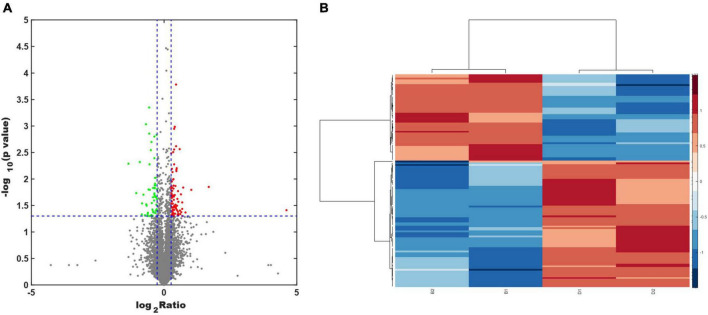
**(A)** Volcano plot analysis of the differentially expressed proteins between the ASE treatment and the PD group. Proteins that have a difference of fold change > 1.2 or < −1.2 and a *P* < 0.05 are defined as significantly different. The red points indicate proteins with a significant upregulation, while the green points indicate proteins with a significant downregulation. **(B)** Heatmap analysis of the differentially expressed proteins between the ASE treatment and the PD group.

**FIGURE 4:**
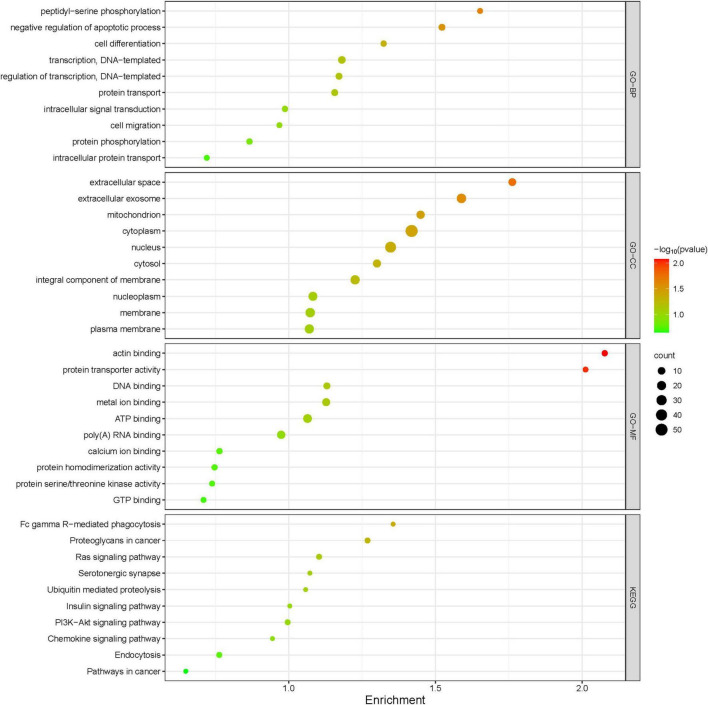
Gene Ontology functional annotations and KEGG pathway analysis of ASE in treating PD. The radius of the circle indicates the number of genes enriched, while the color indicates the number of *p*-values.

Protein extracts from the brains of control, model, and ASE-treated mice were analyzed to learn more about the role of ASE in MPTP-induced PD. ITRAQ was used to label fractions recovered after high-abundance protein was removed, and LC–MS/MS was utilized for detection. The proteomic analysis uncovered the changes in 5,306 different proteins. 128 proteins in the ASE-treated group were significantly different from those in the model group (fold change > 1.2, P < 0.05), with 76 showing up-regulation and 52 showing down-regulation. Figure 3 displays a volcano plot for differential protein screening and the heatmap analysis of the differentially expressed proteins between the ASE treatment and the model group. GO analysis of the differential proteins between the model group and the ASE-treated group is shown in Figure 4. In the biological process, the differentially expressed proteins (DEPs) are mainly concentrated in peptidyl-serine phosphorylation, negative regulation of apoptotic process, and cell differentiation. Cellular component analysis shows that among the differentially regulated proteins in the ASE-treated group, cytoplasmic proteins accounted for 20.1%, nuclear proteins for 13.6%, and extracellular alien proteins for 13.2%. Molecular function (MF) analysis shows that 8.2% of proteins have homodimerization activity, 1.5% have transport activity, and 5.3% have serine/threonine kinase activity. DNA-based transcription (20.0%), protein transport (12.4%), and DNA-based transcriptional regulation (12.3%) rank first, second, and third, respectively, in the list of biological processes in which proteins are involved. The KEGG analysis results of differential proteins showed that the typical pathway corresponding to DEPs between ASE-treated and model groups was associated with Fcγ R-mediated phagocytosis, Ras signaling pathway, PI3K-AKT signaling pathway.

### 3.4. Role of ASE in canonical pathways and networks

**FIGURE 5:**
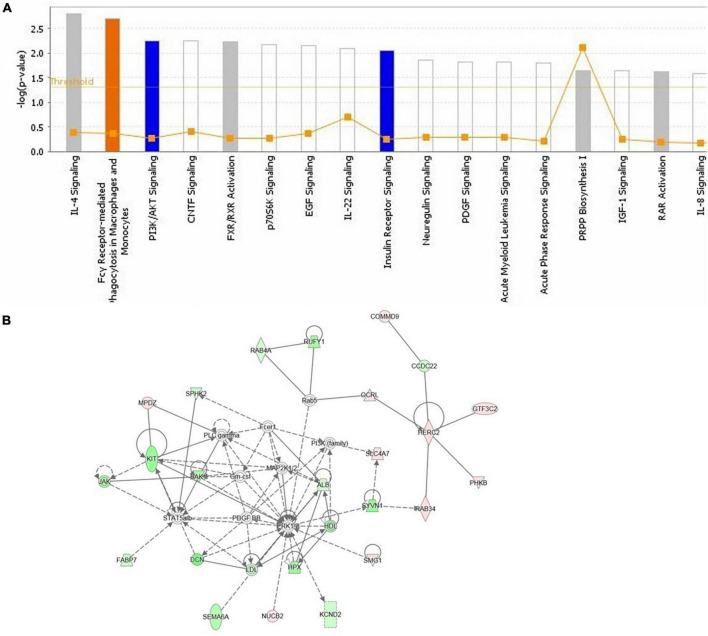
**(A)** The highest ranked canonical IPA pathways of the differentially expressed proteins. The horizontal axis shows the pathway name and the vertical axis is the *p*-value of each pathway. **(B)** The top protein-protein interaction network responding to ASE treatment for PD was predicted by IPA. Twenty-six proteins are involved in this network. The symbols labeled in red and green represent up-regulation and down-regulation, respectively.

Functional annotation and pathway analysis of DEPs were performed to delve deeper into the ASE-related mechanism in the treatment of PD. According to IPA annotation, 128 DEPs responding to ASE treatment play critical roles in organismal injury and abnormalities (n = 117) and neurological disease (*n* = 38), different cell activities like cellular development (n = 40), cellular assembly and organization (*n* = 37), cellular function and maintenance (n = 36), and cellular growth and proliferation (*n* = 36). Protein-protein interaction (PPI) network analysis was performed using IPA, and the results suggested that Fcγ receptor-mediated phagocytosis in macrophages and monocytes, PI3K/AKT signaling, and insulin receptor signaling pathway were the most significantly enriched pathways by ASE treatment (Figure 5A). Ten proteins were found to be up-regulated (red), and sixteen proteins were found to be down-regulated (blue) among the 128 proteins identified as responsive to ASE treatment (green). Thus, cellular morphology, lipid metabolism, and cellular assembly and organization were heavily represented in the most significant PPI network (score = 47) (Figure 5B). 23 key ASE targets for PD therapy were preliminarily identified as ALB, CCDC22, COMMD9, DCN, FABP7, GTF3C2, HERC2, HPX, JAK1, KCND2, KIT, MPDZ, NUCB2, OCRL, PHKB, RAB34, RAB4A, RUFY1, SEMA6A, SLC4A7, SMG1, SPHK2, and SYVN1.

## 4. Discussion

*Acanthopanax senticosus* is a well known traditional Chinese medicine and has been widely used as popular functional food for maintaining and promoting the health. In our previous study on the extract of *Acanthopanax senticosus* (ASE), we found that stress-induced oxidative damage was effectively protected by ASE *in vitro*. And we screened and identified a variety of monoamine oxidase B inhibitors from ASE that may offer therapeutic potential in neurodegenerative diseases (14). To examine whether ASE administration improved locomotor function in MPTP-induced PD mice, we conducted motor behavioral tests including spontaneous activity test, rotarod test, pole-climbing test, and the hanging test. A pre-test was conducted prior to ASE administration to determine the dosage. A low, median, and high dose were set as representatives of 2.25, 4.5, and 13.5 g of crude drug per kg/day. The results showed that the mice in the high-dose administration group showed significant weight loss after ASE administration and almost all died by the end of the 15th day. The behavioral results in the low-dose administration group was not significantly different from that in the control group, indicating that the low-dose group had no obvious effect on anti-PD. In contrast, the mice in the median-dose group showed improved behavior as compared to those in the model group, and their weight and mental state did not differ from those in the model group. Therefore, based on the studies evaluated above, the medium dose (4.50 g crude drug kg^–1^d^–1^) was selected as the administration concentration. Behavioral evaluation of this study indicates that dietary ASE mitigate behavioral deficits in MPTP-induced PD mice. These results confirm and extend our earlier of screening multiple monoamine oxidase inhibitors from ASE.

Furthermore, by using iTRAQ-based proteomics and bioinformatics, we identified 128 proteins that were differentially expressed after ASE administration and predicted multiple signaling pathways. our findings suggest that most of the ASE modulates proteins were involved with Fcγ receptor-mediated phagocytosis in macrophages and monocytes signaling pathway, PI3K/AKT signaling pathway, and insulin receptor signaling pathway. In macrophages and monocytes, the Fcγ receptor controls the expression of PLA2G6, RPS6KB1, AKT1, and PLD1. Fcγ receptors (FcγRs) for IgG are surface glycoproteins that mediate the interaction between antibodies and effector cells, thereby connecting the cellular and humoral arms of the immune system. Some immune system cells, known as effector cells, express FcγRs and are responsible for processes like phagocytosis, inflammatory cell activation, and antibody-dependent cell-mediated cytotoxicity (ADCC) (37). So far, there are 4 distinct FcγRs classes (FcγRI, FcγRIIB, FcγRIII, and FcγRIV) being identified in mice (38, 39). Since ASE appears to regulate Fc receptors, we hypothesize that it can dampen the inflammatory response brought on by PD. In future studies, more research on ASE inhibitors that block the Fcγ receptor-mediated phagocytosis in macrophages and monocytes signaling pathway is needed. Additionally, ASE activates the insulin receptor pathway, which is closely related to insulin resistance. Insulin is not only responsible for glucose balance and energy metabolism as a peripheral hormone. It can also pass through the blood-brain barrier (BBB) and affect many life processes in the brain including regulating the survival and growth of neurons, maintaining synaptic stability and so on (40). A growing number of studies have shown that insulin resistance occurs in the brains of PD patients and animal models. When insulin resistance occurs in the brain, it will cause abnormal aggregation of α-synuclein, mitochondrial dysfunction, neuroinflammation, and cognitive impairments, all of which play a key role in the pathogenesis of PD. Moreover, the aggregation of alpha-synuclein can also inhibit the insulin signaling pathway, further aggravating the disease (41). As a result, insulin-sensitizing drugs such as thiazolidinediones, GLP-1 analogs, and DPP-IV inhibitors may delay PD pathogenesis. In this study, ASE was shown to regulate insulin receptor signaling related proteins. Therefore, we proposed that ASE might increase insulin sensitivity by directly or indirectly increasing insulin synthesis and secretion, which would also contribute to the treatment of PD. Meanwhile, we believe that active ingredients related to insulin receptor signaling pathways can be used as a reference for drug development against PD. Simultaneously, PI3K and AKT are the downstream effector in the insulin receptor substrate (IRS), and when insulin binds to insulin receptors, IRS phosphorylation is induced, and the signaling of the PI3K/AKT pathway is activated. The PI3K/AKT signaling pathways control phagocytosis by regulating RPS6KB1, JAK1, AKT1, and OCRL and thus have important effects on anti-apoptosis and nerve regeneration. Notably, PI3K/AKT pathway activation in PD is found to significantly affect axonal regrowth in adult nigrostriatal projection. A high level of PI3K/Akt pathway activity is associated with neuro-defense, which shows a neuroprotective effect by preventing neuroinflammation and apoptosis (42). It is therefore possible that ASE may exert its neuroprotective effect by activating PI3K and Akt signaling pathways. According to our findings, ASE can simultaneously regulate PI3K/AKT signaling and insulin receptor signaling pathways, both of which are linked to diabetes, suggesting that ASE may be effective in treating PD complicated by diabetes. The findings of our study support the use of ASE as a dietary supplement for chronic disease prevention and management.

## 5. Conclusion

In this study, we used iTRAQ-based quantitative proteomics to look into the effects of ASE on mice with PD and reveal new mechanistic insights of the therapeutic effect of ASE in PD. Our results shed novel light on several protein targets for botanical molecules to alleviate motor deficits. According to our identified protein levels, several target-related pathways were predicted. Our research indicates that the therapeutic mechanism by which ASE fosters resilience in neurological disorders involves its action on multiple targets. It may be possible to better understand the therapeutic mechanisms that promote resilience to neurological diseases by analyzing the multi-target action of ASE.

## Data availability statement

The original contributions presented in this study are included in the article/supplementary material, further inquiries can be directed to the corresponding authors.

## Ethics statement

The animal study was reviewed and approved by Institutional Animal Ethics Committee (IEC) of Jilin University.

## Author contributions

HZ and TZ conceived the idea and designed the experiments. JL, YH, JF, YW, and XF performed the experiments or performed data analysis. JL and YH wrote the manuscript. HZ and TZ revised and edited the manuscript. All authors commented on and approved the manuscript.
